# Preliminary Experience with Extradural Clinoidectomy and Lamina Terminalis Fenestration in Anterior Communicating Artery Aneurysm Surgery: A Matched Case–Control Study

**DOI:** 10.3390/jcm14207413

**Published:** 2025-10-20

**Authors:** Yasmin Sadigh, Joost W. Schouten, Erik H. P. van Putten, Ruben Dammers, Victor Volovici

**Affiliations:** 1 Department of Neurosurgery, Erasmus MC Stroke Center, Erasmus MC University Medical Center, 3015 GD Rotterdam, The Netherlands; 2 Center for Complex Microvascular Surgery, Erasmus MC University Medical Center, 3015 GD Rotterdam, The Netherlands

**Keywords:** intracranial aneurysms, microsurgical clipping, lamina terminalis fenestration, extradural clinoidectomy, anterior communicating artery, shunt-dependent hydrocephalus

## Abstract

**Background/Objectives**: The anterior communicating artery (AcomA) is one of the most common sites of intracranial aneurysms. We aimed to investigate the effect of routine extradural anterior clinoidectomy (EAC) and extradural lamina terminalis fenestration (ELTF) on the incidence of shunt-dependent hydrocephalus (SDH) and gyrus rectus injury in patients undergoing microsurgical clip reconstruction. **Methods**: This matched case–control study included 15 patients treated with routine EAC/ELTF between July 2023 and June 2025, matched 1:2 to 30 historical controls (2000–2019) by aneurysm size, location, dome-to-neck ratio, and rupture status. The primary outcome was the incidence of SDH. The secondary outcomes included the incidence of gyrus rectus hypodensity/injury and clinical outcomes, as assessed by the modified Rankin Scale (mRS) at discharge and follow-up. **Results**: Among 15 cases, 6 had ruptured aneurysms, 4 had unruptured aneurysms, and 5 were recanalized post-endovascular treatment. EAC was performed in all cases; ELTF was performed in 83% of ruptured cases. SDH occurred in 33% of ruptured cases versus 90% in controls (*p* = 0.02). Gyrus rectus hypodensity occurred in 13% of cases vs. 50% of controls (*p* = 0.01). EAC/ELTF was associated with reduced odds of SDH (OR: 0.06; 95% CI: 0.004–0.80; *p* = 0.03) and gyrus rectus hypodensity (OR: 0.15; 95% CI: 0.03–0.80; *p* = 0.03). A poor outcome (mRS >2) was seen in 27% at discharge, improving to 14% at follow-up (with a median of 11 months). Delayed cerebral ischemia occurred in 33% of ruptured cases. **Conclusions**: Routine EAC/ELTF may reduce SDH and gyrus rectus injury after AComA aneurysm clip reconstruction, particularly in ruptured cases. Prospective multi-center studies are needed to validate these preliminary findings.

## 1. Introduction

Aneurysmal subarachnoid hemorrhage (aSAH) is a severe form of hemorrhagic stroke with high rates of morbidity and mortality [1,2]. The anterior communicating artery (AcomA) is one of the most common sites of intracranial aneurysms (IAs). It accounts for a significant proportion (30–40%) of cases presenting with aSAH and up to 15% of cases presenting with unruptured intracranial aneurysms (UIAs) [3]. Since the publication of the International Subarachnoid Aneurysm Trial (ISAT) [4], microsurgical clip reconstruction has been increasingly reserved for larger or anatomically complex aneurysms that are not suitable for endovascular treatment [5].

Achieving optimal surgical exposure to ruptured AcomA aneurysms is often technically demanding due to a narrow subarachnoid space caused by parenchymal swelling and the presence of blood clots. Traditionally, partial resection of the gyrus rectus was performed to improve visualization of the interhemispheric fissure and the A2 segment’s anatomy. To enhance maneuverability and gain a wider exposure to the aneurysmal neck and surrounding anatomy, including improved proximal and distal control, we employ routine extradural anterior clinoidectomy (EAC). EAC is a demanding yet facilitating skull base technique, introduced by Dolenc in 1985 for clip reconstruction of carotid-ophthalmic aneurysms [6].

EAC can be performed via an intradural or extradural approach. The extradural approach preserves dural integrity, reducing the risk of surgical injury to the underlying eloquent structures and minimizing brain traction [6,7,8]. Anatomical variations in the anterior clinoid process (ACP), including an elongated ACP prominence, the existence of a bony carotid ring, a carotico-clinoid foramen, a middle clinoid process forming a bone bridge, and ACP pneumatization, may increase the technical complexity and risk of complications [8,9,10]. The presence of ACP pneumatization increases the potential of cerebrospinal fluid (CSF) leakage. The existence of a carotico-clinoid foramen as an anatomical variation adds to the risk of internal carotid artery (ICA) injury. At our center, EAC has been used routinely in a wide range of anterior skull base pathologies involving the optic apparatus [11]. Since 2023, we have adopted EAC as a standard component in the surgical approach to both ruptured and unruptured AcomA aneurysms, improving visualization while preserving the gyrus rectus.

Access to the CSF is critical for brain relaxation, especially in cases with a high Fisher score, where cisternal CSF is sparse. Lamina terminalis fenestration is an essential CSF-releasing step that has been used routinely in our center for the past 10 years during microsurgical treatment of ruptured IAs. However, this sometimes requires some manipulation and retraction of the frontal lobe. In 2023, we introduced “extradural” lamina terminalis fenestration (ELTF), developed as an extension of the EAC described by Krisht et al. (2022) [12]. After unroofing the optic canal during EAC, the optic sheet is incised with an arachnoid knife parallel to the optic nerve. The incision is then enlarged subfrontally over the optic nerve. The arachnoid dissection is carried posteriorly, following the optic nerve to the lamina terminalis, which can be fenestrated “extradurally”. This technique reduces the risk of frontal lobe injury, particularly in swollen or contused brains.

As a complication of aSAH, hydrocephalus occurs in 6 to 67% of cases [13,14,15,16,17,18]. It may cause shunt dependency, including temporary external ventricular drainage (EVD) or permanent ventriculoperitoneal (VP) shunting, to alleviate intracranial pressure and restore normal CSF dynamics [13]. Previous studies have suggested that lamina terminalis fenestration during microsurgical clip reconstruction of anterior circulation aneurysms may significantly reduce the incidence of shunt-dependent hydrocephalus (SDH) by 4%, with a relative risk of 0.67 (with a 95% confidence interval of 0.50–0.90) of SDH [14]. Favorable clinical outcomes have also been reported more frequently in cohorts undergoing LTF, although analyses were unadjusted [19].

To date, no data are available on the effect of extradural LTF on SDH. Moreover, the evidence of the impact of skull base techniques such as EAC and ELTF on clinical outcomes remains insufficiently studied, with existing evidence limited to unmatched cohort studies with no confounder adjustment [14]. No randomized or matched case–control studies have addressed these techniques specifically [14]. Therefore, the aim of this study was to investigate the effect of routine ELTF and EAC on the incidence of SDH and gyrus rectus injury in patients undergoing microsurgical clip reconstruction of ruptured and unruptured AComA aneurysms, using a matched case–control design.

## 2. Materials and Methods

### 2.1. Eligibility Criteria

All consecutive patients with intracranial aneurysms who underwent microsurgical clip reconstruction between January 2000 and January 2019 were included in a retrospective and partially prospectively maintained master database, published in a previous report [20]. Study cases were identified from a prospectively maintained database from July 2023 to June 2025 and were treated by the senior author (V.V.). These patients were matched in a 1:2 ratio, with controls derived from the master database. The matching criteria included aneurysm location (AcomA), aneurysm size, dome-to-neck ratio (maximum dome diameter/neck width), and rupture status (ruptured or unruptured). Cases involving unruptured recanalized aneurysms were matched with controls with unruptured aneurysms, as no recanalized aneurysms were present in the master database.

General informed consent for data collection and use was obtained from every patient upon admittance to the hospital. Given the retrospective study design and the exclusive use of routinely collected clinical data, the requirement for additional ethical approval was waived by the Erasmus MC Medical Ethics Review Committee. This case–control study was conducted and reported in accordance with the Declaration of Helsinki and the Strengthening the Reporting of Observational Studies in Epidemiology (STROBE) guidelines [21].

### 2.2. Data Collection and Outcome Definitions

Baseline demographic and clinical data, including age at treatment, sex, history of hypertension and smoking, previous aSAH, and the presence of other intracranial aneurysms, were collected from electronic medical records. Anatomical characteristics included aneurysm size (very small (<6 mm), small (6–10), large (11–25), and giant (>25 mm)), the aneurysm dome-to-neck ratio, and aneurysm projection (anterior, superior, or inferior) [22]. The presence of anatomical variations, such as a pneumatized anterior clinoid process, carotico-clinoid foramen, or clinoid bar, was assessed using Ultra-High-Resolution Photon-Counting Computed Tomography Angiography (UHR-PC-CTA).

Data regarding the clinical presentation were assessed using the baseline modified Rankin score (mRS) [23], the World Federation of Neurosurgical Societies (WFNS) grading scale [24], the modified Fisher grade [25] for ruptured aneurysms, and the presence of intracerebral hemorrhage (ICH).

Treatment-related data were also collected, including whether endovascular treatment was attempted prior to surgery, whether standard ELTF and EAC were performed, the number of temporary clipping sessions, and the total duration of temporary clipping (in minutes). Postoperative outcomes such as complete aneurysm occlusion, confirmed by CTA, mRS at discharge, mRS at the first and last follow-up, the length of follow-up, and aneurysm recurrence were extracted. The mRS assessments were constructed based on a detailed and routine neurological assessment performed by clinicians at baseline and follow-up [26] and were checked by the senior author (V.V.). A poor clinical outcome was defined as an mRS of >2 at discharge and follow-up.

Postoperative complications were categorized as the occurrence of SDH and the rate of detected gyrus rectus injury, assessed as either increased hypodensity in postoperative CT or new encephalomalacia in postoperative MRI. Assessment of gyrus rectus injury was performed by two authors independently (Y.S. and V.V.), blinded to the outcome, and was checked by the senior author (V.V.). We evaluated the presence of vasospasm (established in CTA) and the diagnosis of delayed cerebral ischemia (DCI, established in NCCT). We collected data regarding procedure-related morbidity (permanent postoperative neurological deficits, unresolved after six months of follow-up) and procedure-related mortality. All data for the cases were extracted by the first author (Y.S.) and independently reviewed by the senior author (V.V.).

In the early period, EVDs were placed more liberally in our center, when hydrocephalus was observed in CT scans and when it was considered causal for deterioration of consciousness. In the later phase, strict criteria for shunt placement existed in a protocol, with EVDs being indicated by wide ventricles in the admission scan or increasing ventricles in consecutive imaging with a deterioration of consciousness. Patients with intraparenchymal hemorrhage would be considered primarily for microsurgical clip reconstruction. After circa 5 days, if there was a stable clinical situation and the CSF color returned progressively to normal, without signs of massive intraventricular hemorrhage, the EVD was weaned. If there was a deterioration of consciousness, CSF was drained for another 72 h. This procedure was attempted again, and, if it failed, patients received a shunt. If intraventricular hemorrhage was present, it was followed up in serial CT scans, and we waited for 14–21 days before weaning and deciding to place a shunt.

The primary outcome was the incidence of SDH, defined as any shunt placement due to aSAH-induced hydrocephalus. The secondary outcome was defined as the incidence of gyrus rectus injury.

### 2.3. Statistical Analysis

Descriptive statistics were used to characterize the baseline characteristics. The Shapiro–Wilk test was performed to assess the normal distribution of the continuous parameters. Normally distributed variables were reported as means and standard deviations (SDs). Non-normally distributed variables were reported as medians with interquartile ranges (IQRs). Categorical variables were reported as absolute numbers of cases (Ns) and percentages of the total. Data were stratified per rupture status and for recanalized aneurysms. Cases and controls were compared using the Chi-Square test, Fisher’s exact test, or the Fisher–Freeman–Halton exact test for categorial variables and the Mann–Whitney U test or one-way ANOVA for continuous variables. Effect sizes were reported using odds ratios (ORs) with 95% confidence intervals (95% CIs). Statistical analyses were performed using the SPSS software (IBM SPSS Statistics for Windows, Version 29.0.1.0., Armonk, New York, NY, USA). A two-tailed *p*-value of <0.05 was considered statistically significant.

## 3. Results

### 3.1. Patient Inclusion

**Figure 1:**
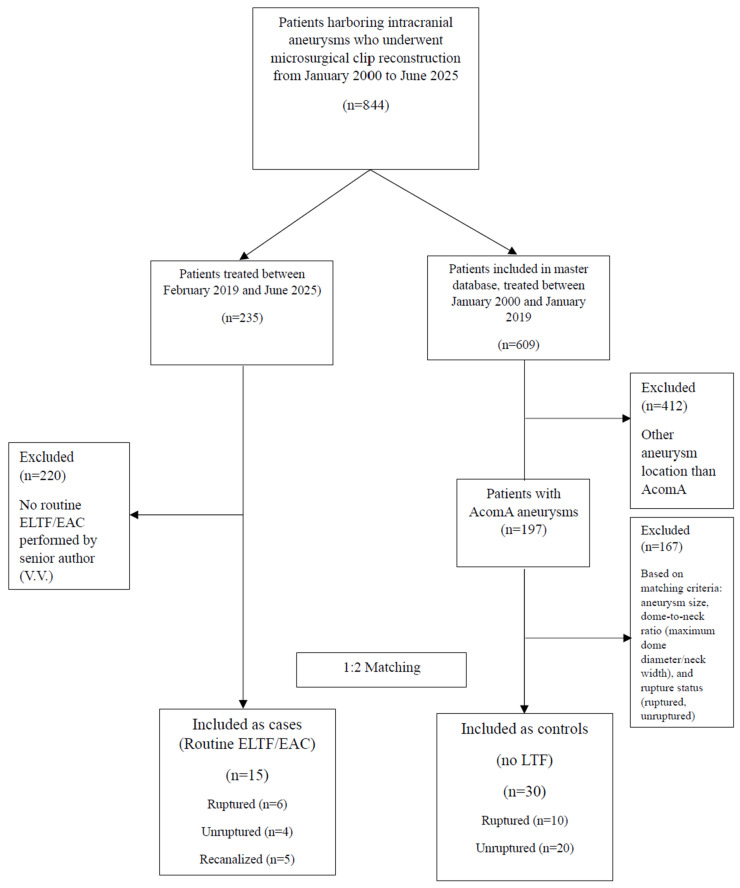
Patient inclusion flowchart for matched (1:2) case–control study. AcomA: anterior communicating artery, ELTF: extradural lamina terminalis fenestration, and EAC: extradural anterior clinoidectomy.

Between January 2000 and June 2025, a total of 844 patients underwent microsurgical clip reconstruction for intracranial aneurysms at our institution (Figure 1).

Of these, 609 patients with 767 aneurysms were included in the master database (containing the data of patients treated between January 2000 and January 2019), and 235 patients were treated between February 2019 and June 2025. Of the patients included in the master database, a total of 412 patients were excluded based on other aneurysm locations than AcomA. In total, 197 patients with AcomA aneurysms from the master database remained. Cases were derived from patients treated by the senior author (V.V.) between July 2023 and June 2025. Ultimately, 15 patients with AcomA aneurysms were included as cases (routine ELTF/EAC) and matched in a 1:2 ratio with 30 controls derived from the master database (no LTF). Of the included cases (*n* = 15), 6 (40%) presented with aSAH, 4 (27%) with unruptured aneurysms, and 5 (33%) with recanalized aneurysms following previous endovascular treatment. One of the recanalized aneurysms presented with aSAH. Cases were matched with 10 controls with ruptured aneurysms and 20 with unruptured aneurysms (Figure 1).

### 3.2. Baseline Characteristics

Of the 15 cases, 8 (53%) were women (Table 1).

The median age was 56 years (IQR: 52–65). Nine (60%) cases had a history of hypertension, and eleven (73%) were smokers. Among the six cases who presented with aSAH, five (71%) presented with a WFNS grade 1 and two (28%) with a WFNS grade 5. In two of these ruptured cases (33%), initial endovascular treatment was attempted but subsequently converted to microsurgical management. One of the recanalized cases presented with WFNS grade I.

In five (33%) cases, the aneurysms had an anterior projection (Table 2).

Seven (47%) cases presented with very small aneurysms (<6 mm). One (7%) case had a pneumatized anterior clinoid process, one (7%) had a clinoid bar, and two (17%) had a carotico-clinoid foramen. As expected, based on the matching design, no significant differences were observed between the case and control groups.

### 3.3. SDH and Gyrus Rectus Hypodensity Outcomes

ELTF was performed in 5 (83%) out of 6 ruptured cases, and EAC was performed in all cases (*n* = 15) (Table 3). In one ruptured case and in the recanalized case presenting with aSAH, it was not documented whether ELTF was performed.

**Figure 2:**
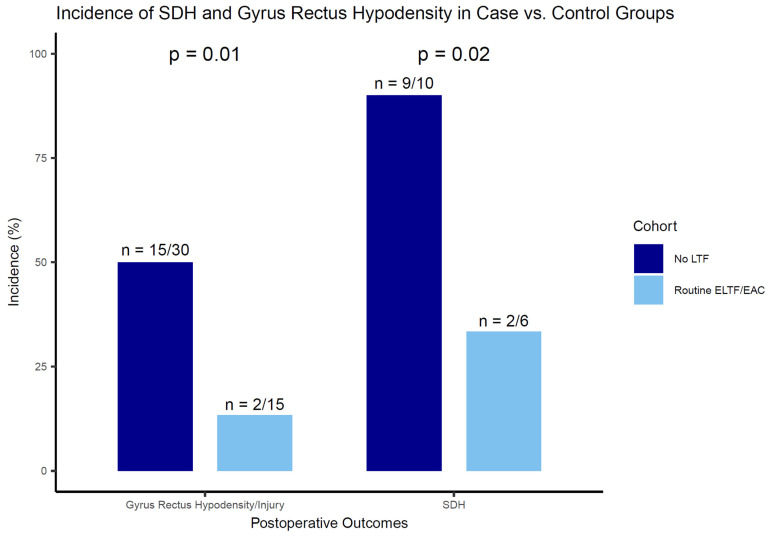
Incidence (%) of SDH and gyrus rectus hypodensity in case vs. control groups. SDH: shunt-dependent hydrocephalus, ELTF: extradural lamina terminalis fenestration, and EAC: extradural anterior clinoidectomy.

The incidence of SDH was 33% (*n* = 2/6) in cases and 90% (*n* = 9/10) in controls with ruptured aneurysms (*p* = 0.02) (Figure 2). Routine ELTF/EAC was associated with a significantly lower incidence of SDH (OR: 0.06; 95% CI: 0.004–0.80; *p* = 0.03).

Gyrus rectus hypodensity occurred in 13% (*n* = 2/15) of cases and in 50% (*n* = 15/30) of controls (*p* = 0.01). None of the cases underwent partial gyrus rectus resection. Routine ELTF/EAC was associated with a reduced incidence of gyrus rectus hypodensity (OR: 0.15; 95% CI: 0.03–0.80; *p* = 0.03).

A demonstration of the routine ELTF/EAC technique is provided in Video S1.

### 3.4. Postoperative and Follow-Up Clinical Outcomes

Postoperative DCI was established in two (33%) cases with ruptured aneurysms (Table 3). One recanalized case (7%) suffered from postoperative ischemia due to vessel occlusion. In all cases, complete aneurysm occlusion was achieved. At discharge, four (27%) patients had a poor clinical outcome. One case died during hospitalization due to pulmonary embolism. A poor clinical outcome at the last available follow-up (a median of eleven months, with an IQR of 9–13) persisted in one patient (14%). The procedure-related mortality was 0%.

### 3.5. Illustrative Case #1

**Figure 3:**
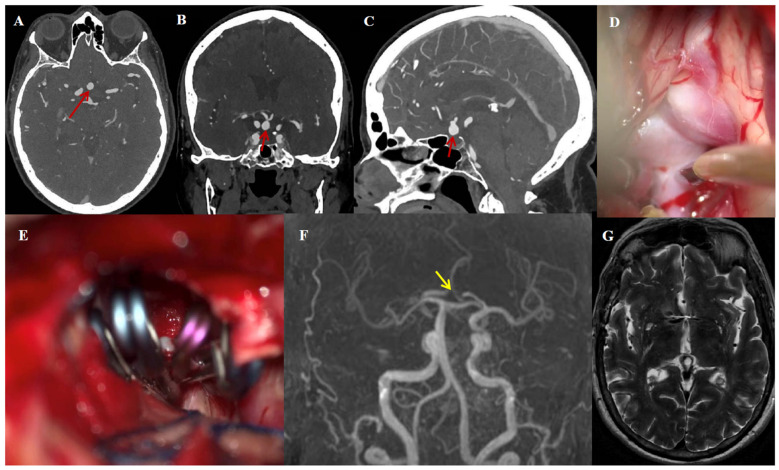
Illustrative case #1. Sixty-eight-year-old woman with incidentally found AcomA aneurysm. (**A**–**C**) Inferior projecting AcomA (8.2 × 6.6 mm; neck size: 3.7 mm) in UHR-PC-CTA (red arrow) in axial, coronal, and sagittal views. (**D**) Anteriorly projecting aneurysm buried in the right optic nerve and chiasm, with a dilated Acom. (**E**) The definitive picket fence clip reconstruction configuration. (**F**) Time-of-flight MRA at nine months of follow-up, demonstrating aneurysm occlusion (no aneurysm observed; yellow arrow). (**G**) At nine months of follow-up, no abnormalities were observed in T2 MRI sequence based on postoperative effects.

A 68-year-old woman with a history of hypertension and migraine presented with transient ischemic attacks (TIAs). An unruptured AcomA aneurysm was incidentally found (Figure 3A–C). Due to the high rupture risk (with a Population, Hypertension, Age, Size, Earlier SAH, and Site [PHASES] score of 3.2%), a supratemporal visual field deficit caused by optic chiasm compression, and a wide aneurysm neck of 3.7 mm, microsurgical clip reconstruction with EAC was indicated (Figure 3D,E). The postoperative course was uneventful, apart from transient oculomotor palsy causing diplopia and cognitive decline, which was resolved during hospitalization and early follow-up. Complete aneurysm occlusion was achieved, confirmed by UHR-PC-CTA postoperatively. After nine months of follow-up, magnetic resonance angiography [MRA] demonstrated no aneurysm recurrence (Figure 3F), and no T2 abnormalities were observed in the surrounding brain parenchyma (Figure 3G).

### 3.6. Illustrative Case #2

**Figure 4:**
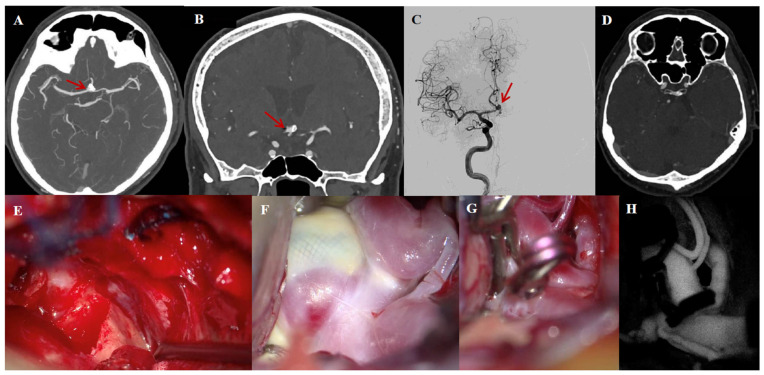
Illustrative case #2. Sixty-seven-year-old male with previously endovascularly treated (WEB) AcomA aneurysm, presenting with a neck remnant. (**A**) Maximum intensity projection neck remnant (red arrow), with undeployed WEB device visible in axial view. (**B**) Coronal view of neck remnant (red arrow), with undeployed WEB device visible. (**C**) Digital subtraction angiography showing neck remnant (red arrow). (**D**) Pneumatized ACP, orbital roof, and lateral orbital wall. (**E**) Removal of the pneumatized clinoid and an unroofed optic canal on the right side. (**F**) The fenestrated Acom, the WEB device, and the still-filling neck remnant. (**G**) The final clip configuration. (**H**) Indocyanine green (ICG) video angiography confirms patency of all normal vessels and exclusion of the aneurysm from circulation.

A 67-year-old man with a history of elective endovascular treatment of an AcomA aneurysm presented with aneurysm rest due to incomplete occlusion following Woven EndoBridge (WEB) deployment (Figure 4A–C). Given a high PHASES score and a wide-neck configuration, microsurgical clip reconstruction with EAC was indicated. An abdominal fat graft was harvested because of ACP pneumatization (Figure 4D) to reduce the risk of CSF leakage following EAC. Complete aneurysm occlusion was achieved (Figure 4E–H), confirmed by CTA postoperatively. The postoperative course was uneventful, and at one month of follow-up, his mRS was 0.

## 4. Discussion

### 4.1. Key Findings

In this high-volume microsurgical series, fifteen patients with AcomA aneurysms underwent microsurgical clip reconstruction with routine EAC/ELTF between July 2023 and June 2025. Cases were matched 1:2 to 30 historical controls treated without ELTF between 2000 and 2019. In the cases (*n* = 15), 40% presented with ruptured aneurysms, 27% with unruptured aneurysms, and 33% with recanalized aneurysms, previously treated endovascularly. EAC was performed in all cases, and ELTF was performed in 83% of the ruptured cases. The incidence of shunt-dependent hydrocephalus (SDH) in ruptured aneurysms was significantly lower in the EAC/ELTF group (33%) compared with controls (90%; *p* = 0.02). Postoperative gyrus rectus hypodensity/injury occurred in 13% of cases versus 50% of controls (*p* = 0.01). EAC/ELTF was associated with reduced odds of SDH (OR: 0.06; 95% CI: 0.004–0.80; *p* = 0.03) and gyrus rectus hypodensity (OR: 0.15; 95% CI: 0.03–0.80; *p* = 0.03). The procedure-related mortality was 0%.

### 4.2. Effect of ELTF on Postoperative SDH

SDH remains a frequent complication of aSAH, with incidence rates ranging from 6% to 67%, depending on the patient population and diagnostic criteria [13,14,15,16,17,18]. In this study, ELTF was associated with a significant reduction in SDH rates in ruptured aneurysms. These findings support prior retrospective cohort studies suggesting a protective effect of ELTF on CSF dynamics and shunt dependency [14]. SDH risk after aSAH is primarily driven by rupture status and the extent of blood clot burden, which contributes to impaired cerebrospinal fluid absorption and flow obstruction [13,14,15,16,17,18]. Therefore, while ELTF may facilitate CSF clearance and reduce the rate of shunting, its effectiveness is inherently influenced by the severity of aSAH and associated inflammatory changes.

While the historical control group and patients from observational studies included patients in whom no LTF was performed and external ventricular drains (EVDs) were used with varying indications, the dramatic difference in SDH rates supports a biologically plausible effect. ELTF is thought to improve CSF flow between the third ventricle and basal cisterns, facilitating the clearance of blood-contaminated CSF and reducing intraventricular pressure. Moreover, aSAH can trigger leptomeningeal inflammation and fibrosis of arachnoid granulations, impairing CSF absorption and contributing to chronic hydrocephalus [27,28,29,30,31,32]. These effects are particularly relevant in high-grade aSAH (Fisher grades 3–4), where the cisternal blood burden is significant [14,15]. ELTF may mitigate this by enhancing acute CSF drainage and modulating the inflammatory cascade.

Importantly, our study is the first to assess ELTF in a matched case–control design, reducing confounding from rupture status and aneurysm morphology. However, the results must be interpreted cautiously due to the small sample size, incomplete follow-up, and non-uniform use of ELTF in all ruptured cases. Further prospective, multicenter, controlled trials using standardized ELTF/EAC techniques are required to validate these preliminary findings and determine the long-term effects on CSF dynamics and patient outcomes [33].

### 4.3. Training, Microvascular Surgical Philosophy, and Skull Base Techniques

The successful and safe implementation of ELTF/EAC in this series reflects a broader paradigm shift in the microsurgical management of complex intracranial aneurysms. Here, advanced microsurgical exposure and intraoperative techniques for CSF drainage were prioritized to optimize outcomes and prevent aSAH-related SDH, especially in complex aneurysms of the anterior circulation. The use of EAC in all cases provided early proximal control, improved exposure of the AcomA complex, and minimized the need for frontal lobe retraction or gyrus rectus resection. In contrast, historical controls often require excessive frontal lobe retraction, increasing the risk of postoperative edema and gyrus rectus injury.

In our institution, EAC has become routine in selected anterior circulation aneurysms, especially those in relation to the optic apparatus [11]. The increased exposure is particularly beneficial in cases with anatomical complexity, such as short A1 segments or AcomA fenestrations, or large, superiorly projecting aneurysms. Navigating the complex bony anatomy of the ACP, including anatomical variations such as ACP pneumatization, the presence of a carotico-clinoid foramen, and clinoid bars, demands not only advanced preoperative imaging (such as UHR-PC-CTA) but also refined anatomical understanding and microsurgical dexterity. Variants such as ACP pneumatization carry a risk of CSF leakage, while bony rings can complicate safe exposure of the ICA [9,10].

Given these challenges, there is a growing consensus that the treatment of ruptured or anatomically complex aneurysms should be exclusively managed in high-volume centers with dedicated multidisciplinary cerebrovascular teams and advanced microsurgical expertise [34,35]. Microsurgical aneurysm management has become increasingly selective in the post-ISAT era [20]. As such, the remaining microsurgical cases are, by definition, more complex and demand a tailored approach requiring subspecialized expertise, which cannot be consistently replicated in low-volume or general neurosurgical units.

### 4.4. Strengths and Limitations

Our study had several strengths. It is one of the first to incorporate a 1:2-matched case–control design to minimize confounding by the rupture status, aneurysm size, and dome-to-neck ratio. Second, this study was conducted in a high-volume tertiary center with standardized surgical techniques.

However, limitations include the small sample size, especially within the ELTF subgroup of ruptured aneurysms, and its retrospective nature. While the controls were selected from a prospectively maintained master database, differences in perioperative care protocols over the 19-year interval may have influenced outcomes. Additionally, EAC and ELTF were adopted as a routine approach only recently (since 2023) and have required a long run-in period, in which the technique has evolved and was refined after the first EAC in 2013 in Rotterdam. Given the recent nature of the cohort, more than half of the cases lacked long-term follow-up data, which precludes definitive conclusions about neurological recovery or late complications, such as delayed hydrocephalus.

Furthermore, the comparison of recent cases with historical controls introduced potential bias due to temporal changes in surgical practices, perioperative management, and patient selection. Moreover, as EAC and ELTF were routinely performed together in our series, the study design did not allow us to distinguish the individual effects of these two techniques on outcomes such as SDH or gyrus rectus injury. Future studies with larger cohorts and more granular study designs are necessary to better elucidate their separate effects. While we observed a lower incidence of gyrus rectus injury in the EAC/ELTF group, the clinical significance of this radiological finding remains uncertain, particularly in the absence of formal neuro-cognitive testing in our study. Future studies with standardized neuropsychological follow-up are needed to clarify the functional relevance of these findings.

Although EAC improves exposure and facilitates proximal aneurysmal control, it remains a technically demanding procedure, not without potential morbidity, including CSF leakage or optic nerve injury, especially in cases with anatomical variants such as ACP pneumatization or the presence of a carotico-clinoid foramen [9,10]. Similarly, ELTF carries risks in ruptured aneurysms, particularly if performed prior to proximal control, where inferiorly projecting aneurysms adherent to the optic chiasm may increase the risk of intraoperative rupture. These risks must be considered when generalizing this approach.

Regarding ELTF, although we observed a significantly reduced rate of shunt-dependent hydrocephalus, the control group’s high shunt rate (90%) exceeded rates reported in the literature, potentially overestimating its benefit. Additionally, the 95% CIs of the effect sizes of both SDH and gyrus rectus injury were large, suggesting statistical uncertainty, which was potentially caused by the small sample size. Moreover, while prior studies and our findings suggest a potential benefit, a meta-analysis by Komotar et al. (2009) questioned the efficacy of lamina terminalis fenestration [36]. However, the interpretation of this lack of effect was influenced by unmatched cohort differences as well as other study limitations included in the meta-analysis. This highlights the need for further prospective, multicenter studies with standardized indications, techniques, and outcome measures.

Finally, while the results support the use of ELTF and EAC in microsurgical clipping of AcomA aneurysms, generalizability may be limited to similarly experienced skull base centers with advanced technical infrastructure and training.

## 5. Conclusions

The routine use of extradural anterior clinoidectomy and lamina terminalis fenestration during microsurgical clip reconstruction of AComA aneurysms may be associated with a reduced incidence of shunt-dependent hydrocephalus and gyrus rectus injury, particularly in ruptured cases. While these preliminary findings suggest potential benefits of incorporating these standardized skull base techniques into microsurgical aneurysm management, they should be interpreted with caution, given this study’s limitations. Larger prospective studies are necessary to confirm these results, evaluate long-term outcomes, and better define the patient populations most likely to benefit.

## Figures and Tables

**Table 1:** Baseline characteristics of matched cases.

No. (%)		All (*n* = 15)	Ruptured (*n* = 6)	Unruptured (*n* = 4)	Recanalized (*n* = 5) *	Missings (%)
Age at treatment, median (IQR)		56 (52–65)	57 (51–72)	60 (54–66.5)	55 (45.5–65.5)	0
Sex, female		8 (53)	4 (67)	2 (50)	2 (40)	0
History of hypertension		9 (60)	3 (50)	4 (100)	2 (40)	0
History of smoking		11 (73)	2 (33)	4 (100)	5 (100)	0
Previous aSAH		-	0 (0)	0 (0)	4 (80)	0
Other intracranial aneurysms		5 (33)	2 (33)	1 (25)	2 (40)	0
Modified Fisher grade	1	-	2 (33)	-	1 (20)	0
	2	-	0 (0)	-	0 (0)	0
	3	-	3 (50)	-	0 (0)	0
	4	-	1 (17)	-	0 (0)	0
WFNS grade	1	-	4 (67)	-	1 (20)	0
	2	-	0 (0)	-	0 (0)	0
	3	-	0 (0)	-	0 (0)	0
	4	-	0 (0)	-	0 (0)	0
	5	-	2 (33)	-	0 (0)	0
ICH		-	0 (0)	-	0 (0)	0
Previous treatment		-	-	-	Coiling (*n* = 4, 80%) WEB (*n* = 1, 20%)	0
Baseline mRS	0	6 (40)	3 (50)	2 (50)	1 (20)	0
	1	6 (40)	1 (17)	2 (50)	3 (60)	
	2	0 (0)	0 (0)	0 (0)	0 (0)	
	3	1 (7)	0 (0)	0 (0)	1 (20)	
	4	0 (0)	0 (0)	0 (0)	0 (0)	
	5	2 (13)	2 (33)	0 (0)	0 (0)	

IQR: interquartile range, aSAH: aneurysmal subarachnoid hemorrhage, WFNS: World Federation of Neurosurgical Societies, ICH: intracerebral hemorrhage, mRS: modified Rankin score, and WEB: woven EndoBridge. * One case presented with a ruptured recanalized aneurysm.

**Table 2:** Aneurysmal characteristics and surgical approach cases.

No. (%)		All (*n* = 15)	Ruptured (*n* = 6)	Unruptured (*n* = 4)	Recanalized * (*n* = 5)	Missings (%)
AcomA aneurysm projection	Anterior	5 (33)	1 (17)	2 (50)	2 (40)	0
	Superior	5 (33)	3 (50)	0 (0)	2 (40)	
	Inferior	5 (33)	2 (33)	2 (50)	1 (20)	
Aneurysm size	Very small (<6 mm)	7 (47)	3 (50)	0 (0)	4 (80)	0
	Small (6–10 mm)	5 (33)	2 (33)	2 (50)	1 (20)	
	Large (11–25 mm)	3 (20)	1 (17)	2 (50)	0 (0)	
Dome-to-neck ratio, median (IQR)		2 (1.4–2.7)	1.8 (1.4–2.7)	2.1 (1.5–3.1)	-	6 (40)
Pneumatized anterior clinoid		1 (7)	0 (0)	0 (0)	1 (20)	0
Clinoid bar		1 (7)	1 (17)	0 (0)	0 (0)	0
Carotico-clinoid foramen		2 (17)	0 (0)	0 (0)	2 (40)	0
ELTF (only in aSAH)		5 (33)	5 (83)	-	-	2 (28)
EAC		15 (100)	6 (100)	4 (100)	5 (100)	0
First-in-line endovascular management attempted		2 (13)	2 (33)	0 (0)	0 (0)	0
Number of temporary clippings, median (IQR)		3 (2–5)	-	3 (2–6)	-	7 (47)
Total duration of temporary clipping, median (min, IQR)		12 (7–26)	-	12 (7–35)	-	8 (53)

IQR: interquartile range, aSAH: aneurysmal subarachnoid hemorrhage, ELTF: extradural lamina terminalis fenestration, and EAC: extradural anterior clinoidectomy. * One case presented with a ruptured recanalized aneurysm.

**Table 3:** Postoperative and follow-up clinical outcomes cases.

No. (%)		All (*n* = 15)	Ruptured (*n* = 6)	Unruptured (*n* = 4)	Recanalized (*n* = 5) *	Missings (%)
Postoperative hypoperfusion gyrus rectus in NCCT		2 (13)	2 (33)	0 (0)	0 (0)	0
Postoperative SDH		2 (13)	2 (33)	0 (0)	0 (0)	0
Vasospasm		4 (27)	4 (67)	0 (0)	0 (0)	0
DCI		2 (13)	2 (33)	0 (0)	0 (0)	0
Postoperative ischemia due to vessel occlusion		1 (7)	0 (0)	1 (25)	0 (0)	0
Aneurysm complete occlusion		15 (100)	6 (100)	4 (100)	5 (100)	0
Discharge mRS	0	1 (7)	0 (0)	0 (0)	1 (20)	0
	1	6 (40)	3 (50)	1 (25)	2 (40)	
	2	3 (20)	1 (17)	2 (50)	0 (0)	
	3	3 (20)	0 (0)	1 (25)	2 (40)	
	4	1 (7)	1 (17)	0 (0)	0 (0)	
	5	0 (0)	0 (0)	0 (0)	0 (0)	
	6	1 (7)	1 (17)	0 (0)	0 (0)	
First FU time, median (mo, IQR)		2 (1–3)	-	2 (1–2)	3 (1–4)	5 (33)
First FU mRS	0	2 (20)	0 (0)	1 (33)	2 (40)	5 (33)
	1	4 (40)	2 (100)	2 (67)	1 (20)	
	2	2 (20)	0 (0)	0 (0)	0 (0)	
	3	2 (20)	0 (0)	0 (0)	2 (40)	
	4	0 (0)	0 (0)	0 (0)	0 (0)	
	5	0 (0)	0 (0)	0 (0)	0 (0)	
Last FU time, median (mo, IQR)		11 (9–13)	-	-	10 (7.5–12.5)	8 (53)
Last FU mRS	0	1 (14)	0 (0)	0 (0)	1 (25)	8 (53)
	1	4 (57)	2 (100)	1 (100)	1 (25)	
	2	1 (14)	0 (0)	0 (0)	1 (25)	
	3	1 (14)	0 (0)	0 (0)	1 (25)	
	4	0 (0)	0 (0)	0 (0)	0 (0)	
	5	0 (0)	0 (0)	0 (0)	0 (0)	
FU aneurysm recurrence		0 (0)	0 (0)	0 (0)	0 (0)	0 (0)
Procedure-related morbidity		1 (10)	0 (0)	0 (0)	1 (20)	5 (33)
Procedure-related mortality		0 (0)	0 (0)	0 (0)	0 (0)	0

IQR: interquartile range, NCCT: non-contrast computed tomography, DCI: delayed cerebral ischemia, SDH: shunt-dependent hydrocephalus, FU: follow-up, mo: months, mRS: modified Rankin score. * One case presented with a ruptured recanalized aneurysm.

## Data Availability

The data are available upon reasonable written request to the corresponding author, including a research proposal and a data-sharing agreement.

## Supplementary Materials

The following supporting information can be downloaded at https://www.mdpi.com/article/10.3390/jcm14207413/s1. Video S1: Demonstration of the routine ELTF/EAC technique.

## Abbreviations

The following abbreviations were used in this manuscript:

aSAH	Aneurysmal subarachnoid hemorrhage
AcomA	Anterior communicating artery
IA	Intracranial aneurysm
UIA	Unruptured intracranial aneurysm
ISAT	International Subarachnoid Aneurysm Trial
EAC	Extradural anterior clinoidectomy
ACP	Anterior clinoid process
CSF	Cerebrospinal fluid
ICA	Internal carotid artery
ELTF	Extradural lamina terminalis fenestration
LTF	Lamina terminalis fenestration
SDH	Shunt-dependent hydrocephalus
EVD	External ventricular drainage
VP	Ventriculoperitoneal
mRS	Modified Rankin score
WFNS	World Federation of Neurosurgical Societies
ICH	Intracerebral hemorrhage
UHR-PC-CTA	Ultra-High-Resolution Photon-Counting Computed Tomography Angiography
DCI	Delayed cerebral ischemia
NCCT	Non-contrast computed tomography
IQR	Interquartile range
PHASES	Population, Hypertension, Age, Size, Earlier SAH, and Site
TIA	Transient ischemic attack
MRA	Magnetic resonance angiography
WEB	Woven EndoBridge
STROBE	Strengthening the Reporting of Observational Studies in Epidemiology
OR	Odds ratio
CI	Confidence interval
